# Characterization of a temperature-responsive two component regulatory system from the Antarctic archaeon, *Methanococcoides burtonii*

**DOI:** 10.1038/srep24278

**Published:** 2016-04-07

**Authors:** T. Najnin, K. S. Siddiqui, T Taha, N. Elkaid, G. Kornfeld, P. M. G. Curmi, R. Cavicchioli

**Affiliations:** 1 School of Biotechnology and Biomolecular Sciences, The University of New South Wales, Sydney, New South Wales, 2052, Australia; 2 Life Sciences Department, King Fahd University of Petroleum and Minerals, Dhahran, Kingdom of Saudi Arabia; 3 School of Physics, The University of New South Wales, Sydney, New South Wales, 2052, Australia

## Abstract

Cold environments dominate the Earth’s biosphere and the resident microorganisms play critical roles in fulfilling global biogeochemical cycles. However, only few studies have examined the molecular basis of thermosensing; an ability that microorganisms must possess in order to respond to environmental temperature and regulate cellular processes. Two component regulatory systems have been inferred to function in thermal regulation of gene expression, but biochemical studies assessing these systems in *Bacteria* are rare, and none have been performed in *Archaea* or psychrophiles. Here we examined the LtrK/LtrR two component regulatory system from the Antarctic archaeon, *Methanococcoides burtonii*, assessing kinase and phosphatase activities of wild-type and mutant proteins. LtrK was thermally unstable and had optimal phosphorylation activity at 10 °C (the lowest optimum activity for any psychrophilic enzyme), high activity at 0 °C and was rapidly thermally inactivated at 30 °C. These biochemical properties match well with normal environmental temperatures of *M. burtonii* (0–4 °C) and the temperature this psychrophile is capable of growing at in the laboratory (−2 to 28 °C). Our findings are consistent with a role for LtrK in performing phosphotransfer reactions with LtrR that could lead to temperature-dependent gene regulation.

Temperature influences the ability of all life on Earth to grow and survive, with the temperature capable of supporting microbial life spanning at least 140 °C, from −20 °C to above 120 °C^1^,^2^,^3^. However, it is noteworthy that individual species have a restricted growth temperature range of ~45 °C or less^4^. This growth temperature limit is caused by cells being constrained by the kinetic and thermodynamic limits of their molecular components^5^,^6^,^7^. For example, proteins from psychrophiles tend to be inherently more flexible than proteins from thermophiles so they can function effectively at low temperature, but this renders them unstable at thermophilic temperatures (and the converse also applies)^5^,^8^. The temperature range and the frequency of temperature variation experienced by microorganisms is also environment specific. For example, surface soil and surface aquatic microorganisms can be exposed to large diel as well as seasonal variation in temperature; pathogens of mammals colonise at controlled body temperatures while also spending periods surviving in the environment at markedly lower temperatures; marine microorganisms can be colonizers of hydrothermal vents exposed to enormous temperature fluxes or be deep-sea pelagic organisms that experience essentially no temperature fluctuations. Therefore the ways in which microorganisms need to respond to temperature varies greatly depending on their ‘lifestyle’.

The cold biosphere contains the largest ‘thermal’ population of life on Earth with ~85% of the global biosphere being at temperatures ≤5 °C^6^,^9^. However, even within a biome like Antarctica where cold prevails, community composition is heavily influenced by local environmental factors which can vary considerably between locations^3^. In view of temperature exerting such a pervasive effect on all life, but also being environment specific, it would be expected that some general and some specific traits of thermal adaptation will have evolved within the vast diversity of microbial life.

A good example of a cellular system that has been linked to the ability of *Bacteria* and *Archaea* to respond to temperature^10^,^11^,^12^,^13^, but also has distinguishing features between these lineages, is the two component regulatory system (TCS)^14^,^15^,^16^,^17^. TCSs are defined by a dimeric sensor kinase (SK) protein that becomes autophosphorylated at a conserved histidine residue in response to an environmental signal, which then transfers a phosphoryl group to a conserved aspartate residue within a receiver domain of a response regulator (RR) protein that in turn regulates gene expression. SKs tend to possess transmembrane domains (TMDs) that anchor them to the membrane thereby providing an extracellular domain (e.g. between two TMDs) that is capable of detecting a specific signal (e.g. ligand such as nitrate). Response to the signal causes a conformational change in the cytoplasmic portion of the SK that modulates its autophosphorylation and phosphotransfer (kinase and phosphatase) abilities. In *Bacteria*, in addition to a receiver domain, RRs tend to possess a helix-turn-helix (HTH) DNA binding output domain^15^,^17^. In contrast, in *Archaea* this output domain is rarely present in RRs and the output domains may instead mediate intracellular trafficking of signals, possibly modulating other classes of transcriptional regulators^16^,^17^. In *Archaea*, TCSs are reported to be more abundant in the genomes of psychrophilic methanogens and mesophiles compared to thermophiles and hyperthermophiles^18^. However, the only biochemical characterization of a TCS from *Archaea* is for the Fil system involved in regulating acetoclastic methanogenesis of the anaerobic sludge methanogen, *Methanosaeta harundinacea*^19^. FilI was shown to autophosphorylate and transfer the phosphoryl group to the RRs FilR1 and FilR2, but phosphatase activity was not examined^19^.

A role for TCSs in functioning as thermosensors and regulating gene expression was originally described for *Escherichia coli*^20^. Subsequent global gene expression and/or gene inactivation studies have linked the role of TCSs to low temperature gene regulation in: animal pathogens including *Bacillus cereus*^21^, *Clostridium botulinum*^22^,^23^*, Edwardsiella tarda*^24^, *Flavobacterium psychrophilum*^25^, *Haemophilus influenzae*^26^, *Listeria monocytogenes*^27^,^28^, *Yersinia pseudotuberculosis*^29^; plant pathogens *Agrobacterium tumefaciens*^10^,^30^ and *Pseudomonas syringae*^31^,^32^,^33^,^34^; environmental bacteria *Bacillus subtilis*^11^,^35^, *Sphingobacterium antarcticus*^31^ and *Synechocystis* sp.^12^; and environmental archaea *Methanococcoides burtonii*^13^ and *Methanolobus psychrophilus*^18^.

Despite the inferred roles of TCSs in thermal regulation of gene regulation, few biochemical studies have been performed examining the molecular basis of thermosensing by SKs^36^,^37^,^38^,^39^. In *P. syringae*, autophosphorylation of the CorS SK has been proposed to occur via temperature-dependent conformational changes that cause the catalytic cytoplasmic region containing the conserved histidine to become sequestered into the membrane, thereby functioning as a thermally sensitive switch^34^. In *E. tarda*, the purified extracellular domain of the PhoQ SK was shown to be temperature sensitive, with secondary structure melting proposed as the mechanism for directly sensing temperature leading to the regulation of genes involved in bacterial virulence^24^. *B. subtilis* DesK^11^ and *Synechocystis* sp. Hik33^12^ are SKs that regulate fatty acid desaturase genes thereby controlling lipid saturation and maintenance of membrane fluidity at low temperature, with the ability of DesK to sense temperature requiring attachment to the cell membrane via its TMDs^35^. In contrast to temperature sensing requiring the tethering of the SK to the membrane, a limited number of studies provide evidence for the cytoplasmic domain being able to function as a thermosensor: *E. coli* Tsr, Tar, Trg, Tap and Aer chemoreceptors^20^,^40^,^41^ and *A. tumefaciens* VirA and Agp1 virulence determinants^10^,^30^.

*M. burtonii* is a psychrophilic methanogen isolated from permanently cold waters of Ace Lake, Antarctica. While the strain is not cultivatable on solid medium and genetic manipulation cannot be performed, *M. burtonii* has served as a useful model for studying cold adaptation^42^. Genome sequence analysis identified an overrepresentation of TCSs indicative of high adaptive potential compared to other methanogens^14^,^43^. A total of 45 TCSs were identified in the *M. burtonii* genome, and all but one (Mbur_0695) of the 14 RRs lacked a HTH output domain^43^,^44^. Mbur_0695 possesses a GlpR type HTH output domain which forms an operon-like structure with the SK Mbur_0694^43^. Proteomic analyses identified higher abundance of the RR, Mbur_0695 in cells grown at low (4 °C) vs high (23 °C) temperature leading to the proposal that the Mbur_0694 (SK) and Mbur_0695 (RR) may form a temperature responsive TCS^13^. Subsequent transcriptomic analyses also showed low temperature regulation of the Mbur_0695 transcript^45^ and both the Mbur_0695 and Mbur_0694 transcripts^46^. In view of its thermal regulation, here we refer to Mbur_0694 as the low temperature responsive sensor kinase LtrK and Mbur_0695 as the low temperature responsive response regulator LtrR.

As no biochemical analyses of TCSs have been performed on psychrophiles (*Bacteria* or *Archaea*) and only one on *Archaea* that did not include an assessment of phosphatase activity^19^, in this study we designed experiments to assess the kinase activity of LtrK (autophosphorylation of the LtrK and phosphotransfer to LtrR) and phosphatase activity of LtrK (dephosphorylation of LtrR). Mutation analyses were performed to assess whether the archaeal proteins possessed properties in common with bacterial analogs. In addition, amino acid replacements were made for several histidine residues other than the conserved histidine in order to evaluate their role in mediating phosphorylation activities. Importantly, the temperature-dependency of activity and stability of LtrK and LtrR was determined in order to assess their capacity to function as physiological thermosensors for this psychrophile. Finally, the findings for the *M. burtonii* TCS system were considered relative to other well studied TCSs to develop a view on how TCSs evolved to perform thermosensing.

## Results

### *In silico* analyses of LtrK and LtrR

**Figure 1:**
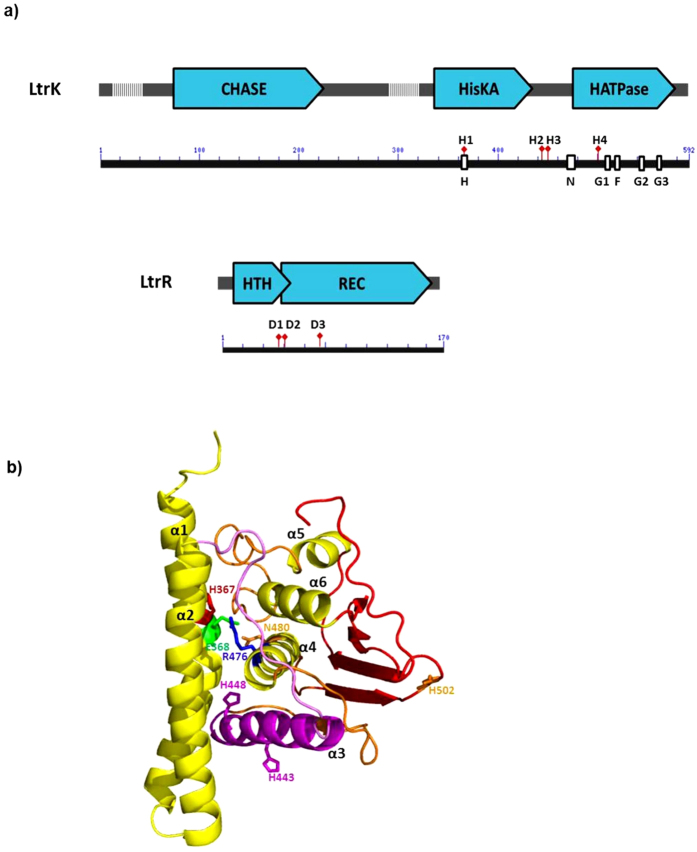
Protein domains and structures predicted for LtrK and LtrR. (**a**) Schematic of LtrK and LtrR protein domains and sequence motifs drawn to scale. Protein domains identified using Pfam and NCBI BLAST (blue arrow boxes); predicted TMDs (hatched regions); H, N, G1, F, G2 and G3 blocks (white boxes) diagnostic of TCS histidine kinases^83^,^84^; specific histidine residues H367 (H1), H443 (H2), H448 (H3), H502 (H4) of LtrK; specific aspartate residues D54 (D1), D55 (D2) and D98 (D3) of LtrR. (**b**) Homology model of the cytoplasmic domain of LtrK constructed using I-TASSER^78^. Only one subunit of the LtrK dimer is shown. The model with the highest confidence score best aligned with the structure of VicK (PDB 4I5S), a TCS SK from *Streptococcus mutans*, which has 37% sequence identity to the cytoplasmic domain of LtrK. The HisKA domain includes the α1 and α2 helices. The α1 helix contains the conserved H367 (red) and E368 (green) residues of the H block. The α4 helix (HATPase domain) contains the conserved N480 (orange) and R476 (blue) residues of the N block. A catalytic triad involved in autophosphorylation^45^,^46^ is formed by R476 (blue), E368 (green) and N480 (orange). The α3 helix (between the HisKA and HATPase domains) contains the additional histidine residues H443 and H448 (magenta).

To identify domains relevant to protein function and residues likely to be involved in phosphorylation, the LtrK and LtrR sequences were compared to TCSs that had been experimentally characterized, including the use of protein homology models constructed from available crystal structures. LtrK was found to possess features typical of dimeric SKs including HisKA (histidine kinase A) dimerization domain and HATPase (histidine kinase like ATPase) domains which form the modular structure of the cytoplasmic domain, as well as a predicted ligand-binding extracellular domain (270 amino acids in length) anchored between two transmembrane domains (TMDs) (Fig. 1a). The cytoplasmic domain of LtrK was compared to three bacterial SKs that have known crystal structures and share at least 35% sequence identity with LtrK (Fig. S1A). Specific motifs within the cytoplasmic domain included: an H block (365-VSH*E*LKTPL-373) which contains a conserved histidine (H367) followed by the motif E/DxxT/N which is diagnostic of the HisKA domain; N (474-LI*R*IFV*N*LLTNA-485), G1 (512-DNGIG-516), F (525-IFDKF-529), G2 (542-GTGLGL-547) and G3 (565-SETGKGS-571) blocks within the HATPase domain; Arg-Asp/Glu-Asn residues from the H and N blocks (italic font within each motif sequence) that form a catalytic triad involved in autophosphorylation^47^,^48^ (Fig. 1b).

In addition to H367, three other histidine residues were identified in the cytoplasmic domain: H443 and H448 in between the H and N blocks in the HisKA and HATPase domains, respectively, and H502 near the G1 block in the HATPase domain (Fig. 1a). Some histidine residues outside of the H block have been shown to function in phosphorylation reactions in some SKs (e.g. *E. coli* NarX and NarQ^49^). However, unlike the additional histidine residues in NarX and NarQ which are in the N block and are conserved in a subfamily of *E. coli* and *B. subtilis* SK sequences^49^, H443, H448 and H502 in LtrK are not conserved in the sequences from the most closely related methanogens *Methanolobus tindarius*, *Methanohalophilus mahii*, *Methanolobus psychrophilus* and *Methanococcoides methylutens* (Fig. S1B). As the *M. methylutens* protein has 75% identity to LtrK across its full length (Fig. S1C), the presence of the additional histidine residues only in LtrK may indicate the histidine residues are not important for kinase/phosphatase activity or they fulfil a function specific to *M. burtonii* LtrK (see **Effects of mutations on phosphorylation activities** below).

The extracellular domain of LtrK contains a CHASE (cyclases/histidine kinases associated sensory extracellular) domain (Fig. 1a). A single CHASE domain is present in some SKs, adenylate or diguanylate cyclases, serine/threonine protein kinases or methyl-accepting chemotaxis proteins from lower eukaryotes and plants, *Bacteria* or *Archaea* and is thought to be a ligand binding domain that enables the sensing of important extracellular signals, including cytokines and short peptides that trigger developmental changes such as spore formation^50^,^51^,^52^. In *Archaea* a specific type of CHASE domain (CHASE4) has been identified that is only present in SKs^51^, including methanogens such as *Methanosarcina acetivorans*^51^ and *M. harundinacea*^19^. However, while LtrK possesses a generic CHASE domain, it does not possess the signature sequences of CHASE4 and may therefore represent an additional class present in *Archaea* (Fig. 1a).

LtrR possesses domains typical of a RR including a HTH domain and a receiver domain (REC) (Fig. 1a). The HTH domain is at the N-terminus of LtrR, whereas the vast majority of RRs in *Bacteria* have the HTH domain at the C-terminus^53^. In *Archaea*, TCSs are mainly represented in methanogens and haloarchaea within the kingdom, *Euryarchaeota*^16^,^53^. From a search of RRs in Integrated Microbial Genomes (IMG), a limited number of RRs with HTH domains were identified in genomes of psychrophilic and mesophilic methanogens, all of which had the HTH domain at the N-terminus (Supplementary Table S1). Genomes of haloarchaea also contained RRs with HTH domains, but ~90% had the HTH domain at the C-terminus, similar to *Bacteria* (Supplementary Table S1). Haloarchaea also tend to possess cold shock proteins (Csps), a class of small nucleic acid binding protein that is the hallmark of *Bacteria*, with very few species of *Archaea* possessing Csps^54^. These data may indicate that within the *Archaea*, psychrophilic and mesophilic methanogens have evolved a class of RRs that has distinct domain architecture to *Bacteria*, and haloarchaea are likely to have acquired Csp genes and most of their RR genes from *Bacteria*.

Fourteen aspartate residues are present in the REC domain of LtrR. Crystal structures of RRs with the highest identity to LtrR are *B. subtilis* PhoP (50% amino acid identity) and *E. coli* PhoB (46%), and the active site aspartate residues in both of them are D10 and D53^55^,^56^. By reference to these structures, the active site aspartate residues in LtrR were inferred to be D55 (or possibly D54) and D98 (Fig. 1a) (see **Effects of mutations on phosphorylation activities** below).

### Overproduction and purification of LtrK and LtrR wild-type and mutant proteins

To obtain a soluble form of LtrK, the N-terminal region encoding the TMDs was deleted and the gene encoding from residue 323 to 592 (Fig. 1) was synthesized with an N-terminal GST tag and overexpressed in *E. coli*. The insoluble fraction (which represented the majority of the protein) was solubilised using Triton X-100 and N-lauroylsarcosine, with subsequent affinity purification generating a pure GST-fusion (~59 kDa) or GST-cleaved (~31 kDa) protein (Supplementary Fig. S2A). Using size exclusion chromatography, the oligomeric state of the purified LtrK was determined to be dimeric (data not shown).

LtrR with an N-terminal His tag remained soluble following overexpression. Affinity purification led to ~20% of the protein eluting with 20 mM imidazole and the remaining ~80% with 100 mM imidazole with the latter exhibiting greater purity (Supplementary Fig. S2B). The LtrR (~20 kDa) was further purified by size exclusion chromatography and determined to be pure by LC-MS/MS (data not shown). GST-tagged LtrK mutants (H367R, H443R, H448R, H502R, H367A and double mutants H443R/H448R and H443A/H448A) and His-tagged LtrR mutants (D54N, D55N and D98N) were purified as for their respective wild-type forms.

### Autophosphorylation and phosphotransfer (kinase and phosphatase)

**Figure 2:**
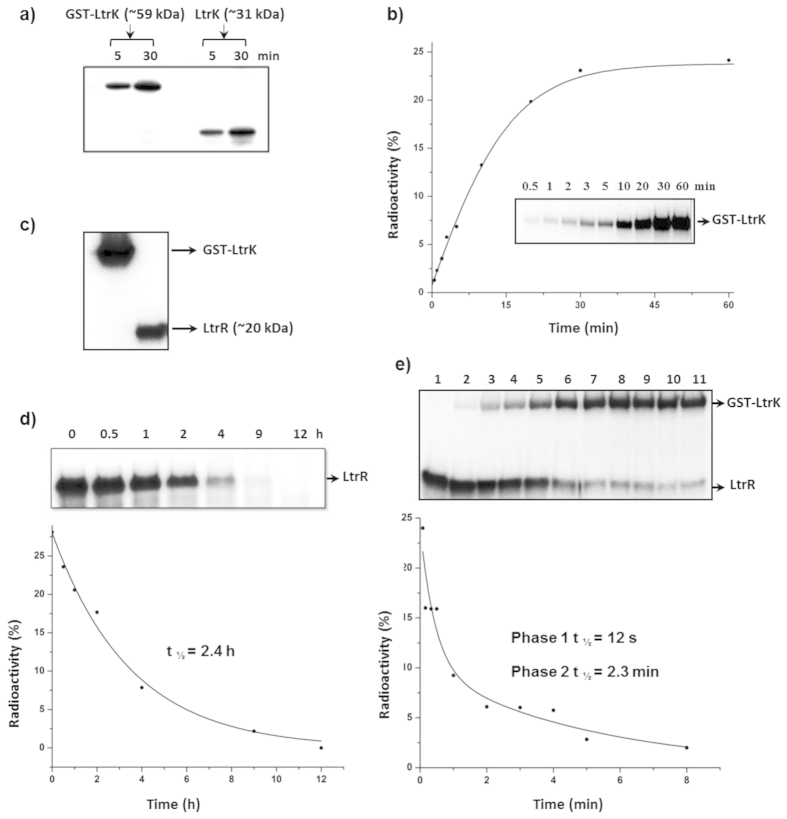
Autophosphorylation and phosphotransfer (kinase and phosphatase) activities of LtrK with LtrR. To assess the phosphorylation state of proteins, samples were electrophoresed on a SDS-polyacrylamide gel, and autoradiography performed by phosphorimaging (panels **a–e**). Incorporation for LtrK and/or LtrR shown as a percentage of the total radioactivity on the respective autoradiograms (panels **b,d,e**). (**a**) Autophosphorylation of LtrK. LtrK fused to GST (GST-LtrK) and LtrK (1 μg) were incubated with [γ-^32^P]-ATP at room temperature. At indicated times, 10 μl samples were added to 5 μl of sample buffer, heated at 95 °C for 3 min and 3 μl of each mixture analysed by gel-phosphorimaging. (**b**) Time course of autophosphorylation. Plot showing autophosphorylation incorporation of GST-LtrK with [γ-^32^P]-ATP over a 60 min incubation at room temperature. The exponential fit curve (solid line) gave a calculated rate constant of 0.08. (**c**) Phosphotransfer from LtrK-P to LtrR. GST-LtrK (15 μg) bound to glutathione agarose beads in a gravity flow column was phosphorylated with [γ-^32^P]-ATP for 30 min at room temperature, free [γ-^32^P]-ATP washed off, LtrR (60 μg) passed through the column and LtrR-P collected in the flowthrough. The LtrR-P sample (10 μl) was added to 3 μl of sample buffer containing 100 mM EDTA, and 3 μl analysed by gel-phosphorimaging. (**d**) Stability of LtrR-P. LtrR-P generated from phosphotransfer from LtrK-P (panel **c**) was incubated at room temperature and retention of γ-^32^P assessed over time (as for panel **b**). The exponential fit curve (solid line) gave a calculated rate constant of 0.29, from which t_1/2_ was calculated as ln2/k. (**e**) LtrK phosphatase activity. As for panel (**d**) except LtrR-P incubated with LtrK. The fit curve (solid line) represents two exponentials with calculated rate constants of 3.45 for the first phase and 0.3 for the second phase 2. Values of t_1/2_ were calculated as ln2/k.

Autophosphorylation assays with [γ-^32^P]-ATP were performed at room temperature with GST-tagged and GST-cleaved forms of LtrK (Fig. 2). Phosphorylation occurred rapidly, reaching a steady state within 30 min (Fig. 2b). The GST tag did not interfere with the autophosphorylation ability of LtrK (Fig. 2b) enabling phosphotransfer experiments to be performed with LtrK immobilized on a GST column (see below).

To investigate whether the autophosphorylated form of LtrK was capable of performing phosphotransfer with LtrR, initially LtrK was phosphorylated with [γ-^32^P]-ATP (LtrK-P) and incubated at room temperature with LtrR and time point aliquots taken and run on an SDS-gel. Phosphorylated LtrR (LtrR-P) was not detected (data not shown) consistent with studies of other SKs which exhibit enhanced phosphatase activity in the presence of [γ-^32^P]-ATP^57^,^58^. To circumvent this, GST-tagged LtrK bound to the GST column was incubated with [γ-^32^P]-ATP, free ATP washed off, and LtrR then passed through the column. This procedure led to γ-^32^P being transferred from LtrK-P to LtrR (Fig. 2c), thereby enabling the elution of LtrR-P from the column to be used for phosphatase assays.

LtrR-P was stable with dephosphorylation occurring with a half-life of ~2.4 h (Fig. 2d), indicating LtrR possessed only weak autophosphatase activity. In contrast, dephosphorylation of LtrR-P was very rapid in the presence of LtrK, and the reaction was biphasic with a half-life for LtrR-P of 12 s in the first phase and 2.3 min in the second phase (Fig. 2e). The biphasic response may occur as a result of dephosphorylation being caused by both autophosphatase activity of LtrR-P and phosphatase activity mediated by LtrK. The loss in ^32^P signal for LtrR-P was also matched by an increasing signal for LtrK-P, demonstrating phosphotransfer from LtrR-P back to LtrK-P (Fig. 2e).

### Effects of mutations on phosphorylation activities

**Figure 3:**
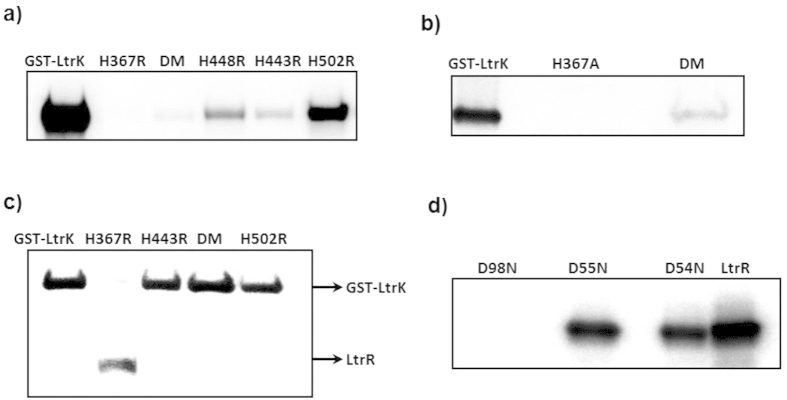
Effects of mutations on the phosphorylation activities of LtrK and LtrR. To assess the phosphorylation state of proteins, samples were electrophoresed on a SDS-polyacrylamide gel, and autoradiography performed by phosphorimaging (panels **a–d**). (**a**) Autophosphorylation of LtrK mutant proteins. Proteins (7 μg) were incubated with [γ-^32^P]-ATP at room temperature for 30 min. Histidine residues were replaced with arginine, including the double mutant (DM) H443R/H448R. (**b**) Autophosphorylation of LtrK mutant proteins. Histidine residues were replaced with alanine, including the double mutant (DM) H443A/H448A. (**c**) Phosphatase activity of LtrK mutant proteins. LtrR-P was incubated with wild-type (lane 1) and mutant proteins H367R (lane 2), H443R (lane 3), H443R/H448R (lane 4), H502R (lane 5) for 30 min at room temperature. (**d**) Phosphotransfer from LtrK-P to LtrR mutant proteins. LtrR (80 μg) wild-type and mutant proteins were phosphorylated by GST-LtrK-P (20 μg) immobilized on a gravity flow column containing glutathione agarose beads as described for Fig. 2c.

All amino acid replacements of histidine led to a reduction in autophosphorylation, but the extent of reduction varied greatly between different mutant proteins (Fig. 3). Histidine was replaced with arginine or alanine to test different structural effects: arginine has similar charge properties to histidine (pH dependent) but has a large side-chain that may disrupt protein structure, whereas alanine has a small, non-polar side-chain, and is less likely to change protein conformation. Only mutations at H367 (both H367R and H367A) completely disrupted autophosphorylation (Fig. 3a,b, lane 2). Mutations H443R and H448R greatly reduced autophosphorylation (Fig. 3a, lane 4, 5), with double mutants H443R/H448R (Fig. 3a, lane 3) and H443A/H448A (Fig. 3b, lane 3) exhibiting even lower activity than the single mutants. However, unlike H367 mutants which had no ^32^P incorporation, a small but detectable level of phosphorylation was observed for these other mutants. The H502R mutation had the least impact on autophosphorylation, although the extent of phosphorylation was less than the wild-type (Fig. 3a, lane 6). The data for autophosphorylation of the mutant proteins are consistent with H367 being the conserved histidine residue that is the site of autophosphorylation, with H443 and H448 being involved in autophosphorylation but not essential for activity. The H443R, H443R/448 R and H502R mutants were all capable of dephosphorylating LtrR-P to a similar extent as the wild-type, whereas H367R was incapable of causing dephosphorylation (Fig. 3c). These data indicate that only H367 and not H443, H448 or H502 are involved in phosphatase activity.

The LtrR mutants D54N, D55N and D98N were assessed for their ability to become phosphorylated by LtrK-P using the phosphotransfer assay (see above). The D98N mutant completely lost the ability to become phosphorylated, whereas the D54N and D55N mutants were as active as the wild-type (Fig. 3d). The data show the essential role of D98 in phosphatase activity and indicate it is likely the residue that accepts the phosphoryl group from LtrK. In addition to the aspartate residue that is the site of phosphoryl-group attachment, two other aspartate residues in the active-site bind a divalent metal ion (typically Mg^2+^) that is essential for RR phosphorylation^59^. As both aspartate and asparagine residues can coordinate Mg^2+^ 60, determining if D54 or D55 function by coordinating Mg^2+^ would require the construction of additional mutants (i.e. cannot be assessed with D54N and D55N).

### Low temperature-dependency of kinase and phosphatase activity

**Figure 4:**
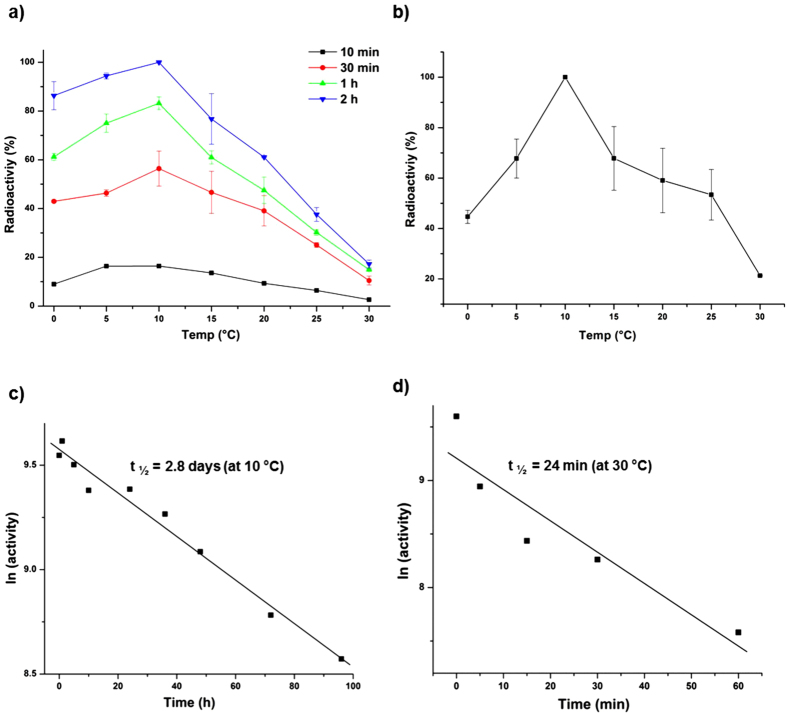
Effect of temperature on kinase and phosphatase activities of LtrK. To assess the phosphorylation state of proteins, samples were electrophoresed on a SDS-polyacrylamide gel, and autoradiography performed by phosphorimaging (panels **a,b**). (**a**) Effect of temperature on autophosphorylation. Autophosphorylation was performed (see Fig. 2b) at different temperatures (0, 5, 10, 15, 20, 25, 30 °C) with aliquots withdrawn for analysis at different times of incubation (10 min, 30 min, 1 h, 2 h). Incorporation is shown as a percentage of the highest band intensity on autoradiograms across all samples (2 h at 10 °C). The mean values for two replicates are plotted for 30 min, 1 h and 2 h, and values for a single time course for 10 min. Error bars represent the standard error of the mean. (**b**) Effect of temperature on phosphatase activity. LtrR-P was incubated with LtrK in a 2 to 1 ratio for 10 min at different temperatures (0, 5, 10, 15, 20, 25, 30 °C) and the band intensity of LtrK-P plotted as a percentage of the highest band intensity on the autoradiograms (LtrK-P at 10 °C). The mean values for two replicates are plotted. Error bars represent the standard error of the mean. (**c**) Half-life of inactivation at 10 °C. LtrK was incubated at 10 °C for up to 4 d and residual autophosphorylation activity determined by incubating aliquots of the enzyme with [γ-^32^P]-ATP for 10 min at 10 °C (T_opt_). The natural log (ln) of activity (band intensity) was plotted against incubation time. The straight line represents the linear fit to the data and the slope of the line was used to calculate t_1/2_ (see **Methods**). (**d**) Half-life of inactivation at 30 °C. As for panel (**c**) except LtrK was incubated at 30 °C.

To determine the optimum temperature (T_opt_) for kinase activity, autophosphorylation assays were performed at 0, 5, 10, 15, 20, 25 and 30 °C for 10 min, 30 min, 1 h and 2 h (Fig. 4a). Autophosphorylation T_opt_ was 10 °C, with considerably higher activity demonstrated after 2 h of incubation at 0 °C compared to 30 °C. In fact, more autophosphorylation occurred after 30 min incubation at 0 °C compared to 2 h incubation at 25 or 30 °C. By assessing activity after only 10 min incubation it also ensured the thermal inactivation of the enzyme was minimized, and that the determination of apparent T_opt_ was therefore performed appropriately^61^. The activity profiles for all temperatures tested show that autophosphorylation was highest at 10 °C (Fig. 4a). A limited analysis of the temperature dependence of phosphoryl group transfer from LtrK-P to LtrR was also performed (Supplementary Fig. S3); ^32^P incorporation was ~2-fold higher at 0 °C compared to 25 °C.

Temperature-dependent phosphatase activities were performed by incubating LtrK with LtrR-P for 10 min at 0, 5, 10, 15, 20, 25 or 30 °C. The phosphatase temperature profile was similar to the kinase temperature profile with T_opt_ at 10 °C, considerable phosphatase activity at 0 °C, and relatively little at 30 °C (Fig. 4b).

The low T_opt_ and time-dependent reduction of kinase and phosphatase activity at 30 °C (Fig. 4a) is suggestive of the enzyme rapidly becoming thermally inactivated. To assess this, the half-life of inactivation (t_1/2_) was determined at 10 °C (T_opt_) and 30 °C (Fig. 4c,d). LtrK was very stable at 10 °C with a t_1/2_ of 2.8 d, whereas at 30 °C t_1/2_ was 24 min. These data indicate that that temperature-dependent loss of activity of LtrK is due to its thermal instability.

**Figure 5:**
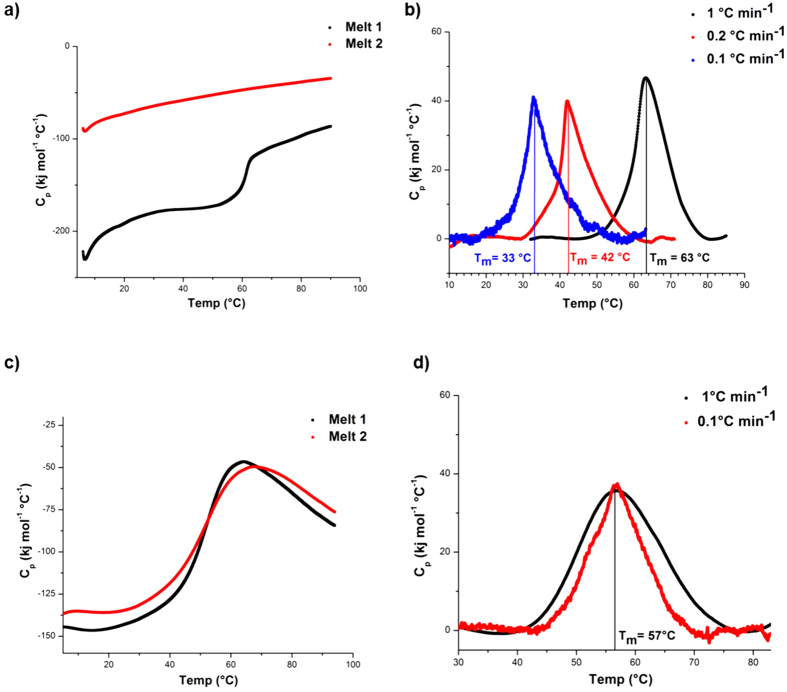
Thermal stability of LtrK and LtrR assessed by DSC. (**a**) Irreversible thermal unfolding of LtrK before baseline correction. Two melts are shown using a scan rate of 1 °C min^−1^. (**b**) Irreversible thermal unfolding of LtrK after baseline correction. The apparent T_m_ for LtrK calculated from DSC thermograms was scan rate dependent. (**c**) Reversible thermal unfolding of LtrR before baseline correction. Two successive melts are shown using a scan rate of 1 °C min^−1^. (**d**) Reversible thermal unfolding of LtrR after baseline correction. The T_m_ calculated for LtrR from DSC thermograms was scan rate independent.

In order to assess the structural basis for the thermally induced loss of kinase and phosphatase activity, biophysical measures of unfolding and stability were performed on both LtrK and LtrR. Differential scanning calorimetry (DSC) thermograms of LtrK showed that the protein unfolded irreversibly (Fig. 5a). Melting temperature (T_m_) was scan-rate dependent: T_m_ 63 °C at 1 °C min^−1^; 42 °C at 0.2 °C min^−1^; 33 °C at 0.1 °C min^−1^ (Fig. 5b). The scan-rate dependency is typical for irreversibly unfolding proteins that are under kinetic control^62^,^63^, reflecting a gradual progression of unfolding over time that accelerates at higher temperatures. By contrast, LtrR unfolded reversibly (hence, independent of scan-rate) with a considerably higher T_m_ (57 °C; Fig. 5c,d) than LtrK (33 °C at a scan-rate of 0.1 °C min^−1^). Moreover, the LtrK DSC thermogram (scan-rate 0.1 °C min^−1^) showed that the protein had already begun unfolding at ~20 °C (Fig. 5b). The fact that the T_opt_ for activity of LtrK (10 °C) is lower than the apparent T_m_ (33 °C at 0.1 °C min^−1^) may reflect the active-site being more heat labile than the main protein structure (i.e. local vs global stability); a feature observed for many psychrophilic proteins^8^,^61^,^64^. Overall, the biophysical data are consistent with the kinetic data and indicate that LtrK is very temperature labile, possessing an inherently unstable structure (possibly the active-site) that unfolds at relatively low temperature and confers relatively high kinase and phosphatase activities at 0 °C but very low activity at 30 °C.

## Discussion

The study provides the first experimental data for the complete phosphorylation cycle of a TCS from *Archaea* as well as for a psychrophile. Achieving this provides the first opportunity to assess what characteristics are typical of well-studied bacterial systems, and what features of the *M. burtonii* TCS are unique and/or appear particularly relevant to temperature-responsive activity, and therefore to speculate about mechanisms that may be involved in thermosensing.

### Characteristics of the *M. burtonii* TCS

Kinase and phosphatase activities of LtrK were retained in the truncated cytoplasmic form of the protein, as has been observed for many bacterial SKs^58^,^65^,^66^. The conserved histidine of LtrK, H367 was found to be essential for both autophosphorylation and phosphatase activities (Fig. 3a,c), similar to *E. coli* EnvZ^67^ and *S. typhimurium* PhoQ^68^, but contrasting with *B. subtilis* DesK or *E. coli* NRII where the conserved histidine is not essential for phosphatase activity^69^,^70^. The stability of LtrR-P (half-life 2.2 h; Fig. 2d) was similar to *E. coli* OmpR (1.5 h)^57^ or *B. subtilis* PhoP (2.5 h)^58^, and much higher than for many bacterial RRs, such as *E. coli* CheY (<5 s)^71^ or *Salmonella typhimurium* NtrC (~5 min)^72^. While some bacterial SKs do not exhibit phosphatase activity, such as *E. coli* CheA^73^ and *S. typhimurium* NtrB^72^, LtrK had very high activity, reducing the half-life of LtrR-P 60–720-fold (biphasic response) and catalysing transfer of the phosphoryl-group back to itself (Fig. 2e).

Perhaps the most interesting findings from the mutation analyses relate to the additional histidine residues. Despite the residues not being conserved in related methanogen sequences including closely related *M. methylutens* (Supplementary Fig. S1B,C), the H443 and H448 mutants had reduced autophosphorylation activity but retained phosphatase activity, including the ability to transfer the phosphoryl group from LtrR-P to LtrK (Fig. 3c). Phosphotransfer activity requires the SK to interact specifically with its cognate RR; the process involving the interaction of dimerization helices in the HisKA domain, docking of the dimer with the RR and transfer of the phosphoryl group from the SK to the RR^47^,^48^. In the protein homology model of the cytoplasmic domain of LtrK, H443 and H448 are in the α3 helix that is positioned between the HisKA and HATPase domains, connected to the HATPase domain via a β-strand to the α4 helix containing the N block (Fig. 1a). The N and G2 blocks within the HATPase domain interact with ATP to position the γ-phosphoryl group near the conserved histidine in the HisKA domain^47^,^48^. Mutation studies targeting the α3 helix do not appear to have been reported in the literature. Our data for H443 and H448 suggest that the α3 helix in LtrK may function by facilitating interactions with ATP to assist in catalysis leading to autophosphorylation. As these histidine residues are not generally conserved in SKs (Supplementary Fig. S1), their function may be specific to interactions between LtrK and ATP. It is also possible that they contribute to the activity and/or stability properties of LtrK.

### Temperature dependency of LtrK

By surveying 33 different proteins purified from psychrophiles including two from *Archaea*, four from *Eucarya* and 27 from *Bacteria*, the T_opt_ was found to range from 16–64 °C with an average of ~36 °C ± 12 (Supplementary Table S2). The 10 °C T_opt_ of LtrK is therefore low, even for an enzyme from a psychrophile. The only report of T_opt_ lower than 16 °C (Antarctic marine bacterium DNA ligase^61^) was 10 °C for nitrate reductase activity from a whole cell extract of a psychrophilic green alga^74^; activity that could reflect multiple gene products and isozymes, and be influenced by other cellular components present in the extract. The fact that enzymes from psychrophiles tend to have a high T_opt_ relative to the environmental temperature of the organisms from which they are derived, results from the kinetic effect of temperature leading to faster reaction rates at higher temperatures; a fact that also leads to relatively high T_opt_ values for the psychrophilic microorganisms themselves^5^,^7^,^42^. *M. burtonii* is capable of growth in the laboratory between −2 °C and 28 °C, and has been found to be heat stressed at temperatures between 23 °C and 28 °C, cold stressed at −2 °C, and cells ‘happily’ growing at 1–16 °C^4^. The high activity of LtrK at 0–10 °C (Fig. 4a,b), short half-life of inactivation at 30 °C (Fig. 4d) and irreversible unfolding at relatively low temperature (~20 °C, Fig. 5b) match well with the environmental temperatures (0–4 °C) *M. burtonii* is exposed to in Antarctica. The findings are consistent with the TCS fulfilling a physiological role in regulating gene expression in response to growth temperature.

### Temperature responsive TCSs in *Archaea* and *Bacteria*

**Figure 6:**
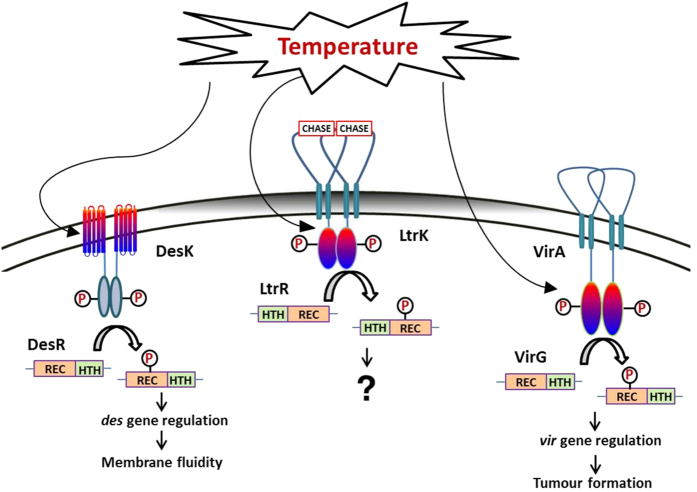
Schematic representation of possible thermosensing by specific SKs from *Archaea* and *Bacteria*. DesK/DesR from *B. subtilis*. DesK senses temperature through its TMDs which manifest as coiled-coil interactions of the DesK homodimer^35^, thereby regulating the phosphotransfer activities of DesK with DesR. DesR regulates the expression of desaturase genes which function to control membrane fluidity. LtrK/LtrR from *M. burtonii*. The cytoplasmic domain of LtrK has high activity at 0 °C, highest activity at 10 °C and minimal activity at 30 °C, consistent with LtrK/LtrR being able to regulate gene expression in response to growth temperature. LtrK also possesses an extracellular CHASE domain which is likely to detect an environmental signal (other than temperature). LtrR has an N-terminal HTH domain, typical of *Archaea*, which is in the opposite orientation to HTH domains from most *Bacteria*. Also typical of *Archaea*, *M. burtonii* possesses ether-linked isoprenoid lipids attached to a glycerol-1-phosphate backbone (membrane shaded grey), whereas *Bacteria* contain ester-linked fatty acids attached to a glycerol-3-phosphate backbone (shaded white). *M. burtonii* lipid unsaturation increases at low growth temperatures^78^. The genes regulated by LtrR have not been determined. VirA/VirG from *A. tumefaciens*. VirA senses temperature via its cytoplasmic domain^10^. Distinct structural domains of VirA are also capable of directly or indirectly detecting sugars, phenolic compounds and acidity levels^76^. VirG regulates genes involved in plant tumour formation.

It is surprising how little is known about the molecular basis of thermosensing by TCSs, given the extent of published experimental data about TCSs and the inferred roles of TCSs in thermosensing (see **Introduction**). Experimental data for thermosensing of SKs is limited to knowledge of the *B. subtilis* DesK/DesR fatty acid desaturase TCS^11^ and *A. tumefaciens* VirA/VirG virulence TCS^10^. The temperature range of activity and stability of LtrK indicates *M. burtonii* may also possess a TCS that is responsive to environmental temperature (Fig. 6).

Autophosphorylation and phosphotransfer of DesK was shown to be higher at 25 °C compared to 37 °C^35^. However, the activity of the cytoplasmic portion of DesK was equivalent at 25 °C and 37 °C, and temperature responsiveness was reported to be dependent on attachment of DesK to the membrane via its TMDs^35^. Temperature-dependent activity was attributed to coiled-coil interactions of the DesK homodimer^35^,^75^, similar to temperature sensitive monomer (unfolded) to coiled-coil (folded) transitions described for the TlpA gene regulator of *S. typhimurium*^36^.

In contrast, the cytoplasmic domain of SKs from *A. tumefaciens*, VirA^10^ and Agp1^30^, are temperature-responsive. The autophosphorylation of the purified soluble cytoplasmic portion of VirA and phosphotransfer to the RR VirG was highest at 28 °C, reduced at 32 °C and negligible at 37 °C^10^. The findings for Agp1 were similar with autophosphorylation highest at 25 °C, weak at 35 °C and negligible at 40 °C^30^.

Overall, DesK, VirA and LtrK are responsive across temperature ranges that are physiologically/ecologically relevant to each of them: the relatively low-temperature dependent expression of virulence genes controlled by VirA parallels temperatures at which crown gall disease is caused by *A. tumefaciens*; the temperature responsive range of DesK matches the temperature at which cellular changes occur to lipid saturation and membrane fluidity in *B. subtilis*; LtrK functions across the growth temperature range of *M. burtonii* with high activity occurring at Antarctic lake temperatures. The functional temperature range for these SKs is consistent with TCSs that facilitate the growth of the environmental microorganisms at natural environmental temperatures, and is indicative of evolutionary selection for enzymes with specific temperature-dependent properties.

### Environmental signalling and regulation of phosphotransfer activity

While the data show that the cytoplasmic domain of LtrK is inherently thermosensitive and sufficient for kinase and phosphatase activities, it is relevant to also consider the role that the extracellular ligand-binding domain might play in environmental signalling, and how regulation of kinase vs phosphatase activity is controlled in the cell. A limited number of SKs have been shown to respond to both temperature and other environmental signals, including *E. coli* chemoreceptors Tsr, Tar, Trg, Tap and Aer^20^,^40^,^41^, *E. tarda* PhoQ^24^ and *A. tumefaciens* VirA^10^,^76^. For VirA, in addition to temperature regulation by the kinase domain, the linker domain separating the second TMD and the kinase domain senses phenolic compounds and acidity, and the extracellular domain senses sugars via a periplasmic galactose binding protein^76^. These findings illustrate the capacity for SKs, and hence possibly LtrK, to sense additional signals.

As the extracellular domain of SKs is ligand specific, their sequences tend to be unique and shared motifs are not commonly observed. Mutation analysis of motifs that do exist, such as a 17 amino acid ‘P-box’ element in NarX and NarQ, determined that they function in enabling ligand (nitrate/nitrite) responsive gene regulation^77^. As such, the CHASE domain in LtrK is conspicuous as a likely ligand binding-site although it is not apparent what specific ligand it binds (Fig. 6). In this regard it is noteworthy that *M. harundinacea* and *M. burtonii* are both methanogens, and the SK from *M. harundinacea* that contains a CHASE4 motif, FilI, is involved in regulating methanogenesis^19^. However, *M. harundinacea* is limited to growth on acetate, which *M. burtonii* cannot utilize, and *M. burtonii* can grow using trimethylamine and methanol, which *M. harundinacea* cannot utilize. Therefore at best we can conclude that it is likely that the LtrK CHASE domain detects an important, but presently unknown extracellular signal, and regulates the activity of the cytoplasmic domain, which itself responds directly to temperature.

It is also possible that LtrK responds to changes in membrane structure and lipid composition. Archaeal lipids are fundamentally different to bacterial lipids as they contain a glycerol-1-phosphate backbone attached to ether-linked isoprenoid lipids, whereas bacteria contain ester-linked fatty acids attached to a glycerol-3-phosphate backbone (Fig. 6). However, similar to bacteria, lipid unsaturation increases at low temperature in *M. burtonii*; for example, the unsaturated lipids archaeol phosphatidylinositol and hydroxyarchaeol phosphatidylinositol increase from ~14% during growth at 23 °C to ~28% at 4 °C^78^. While the mechanism producing unsaturation in *M. burtonii* does not involve a desaturase, as is the case for *B. subtilis*^11^,^35^ and *Synechocystis* sp.^12^, LtrK may be responsive to membrane structure changes and regulate the genes (e.g. geranylgeranyl reductase) involved in selective saturation^78^. This is an attractive model because the state of membrane fluidity arising from changes in lipid saturation could possibly regulate kinase vs phosphatase activities as it does in *B. subtilis*^11^.

In summary, we hypothesize that ligand detection via the CHASE domain and/or protein conformational changes linked to LtrK being anchored to the membrane regulate the balance between kinase and phosphatase activities of the cytoplasmic portion of LtrK, with the level of phosphorylation of LtrR being dictated by the temperature-dependent activity of LtrK (Fig. 6).

## Methods

### Cloning, overexpression and protein purification of LtrK and LtrR

A gene encoding the cytoplasmic domain of LtrK (from 323 to 592 amino acid) was commercially synthesized in plasmid vector pReceiver-B03 (GeneCopoeia) to produce plasmid pReceiver-B03-LtrK with an N-terminal GST tag and a Tev protease cleavage site positioned for cleavage of the GST tag. *E. coli* BL21 (DE3) (Novagen) served as the host for overexpression of the GST-LtrK protein. A single colony of transformed cells was picked and inoculated into 20 mL of LB media containing ampicillin (100 μg ml^−1^), the culture grown at 37 °C and 220 rpm overnight, 20 mL inoculated into 2 L of the media, and the cells grown at 37 °C and 220 rpm until the optical density at 600 (OD_600_) reached 0.7. Induction of gene expression was performed by adding 0.4 mM final concentration isopropyl β-D-1-thiogalactopyranoside (IPTG; GoldBio) and growth continued for another 3 h, with cells harvested by centrifugation at 4 °C at 11,000 × *g* for 30 min and the cell pellet stored at −80 °C. Frozen cell pellets were thawed and suspended in 10 ml of sonication buffer (50 mM HEPES pH 8.0, 50 mM KCl, and 20% glycerol), lysozyme added to a final concentration of 100 μg ml^−1^, and the mixture incubated on ice for 15 min. Immediately prior to sonication, DTT (Promega) and N-lauroylsarcosine sodium salt (Sigma-Aldrich) were added to a final concentration of 5 mM and 1.5%, respectively, and cells sonicated on ice using a Branson digital sonifier with an 0.5 s pulse at 50% amplitude for a total time of 1 min. The protein containing supernatant was collected by centrifugation at 23,000 × *g* for 30 min at 4 °C. Triton X-100 was added to a final concentration of 2%, with sonication buffer added to achieve a final concentration of N-lauroylsarcosine sodium salt of 0.7% and a total volume of 20 ml. The mixture was incubated on ice for 1 h and then applied to a 1 ml bed volume Pierce Glutathione Spin Column (Thermo Scientific) that was pre-equilibrated with sonication buffer. The column was washed to remove unbound proteins and the bound GST-LtrK eluted with elution buffer (50 mM HEPES pH 8.0, 50 mM KCl, 20% glycerol, 20 mM reduced L-glutathione (Sigma-Aldrich)). The fractions containing the protein were identified using SDS-PAGE and the protein concentrated using Amicon Ultra centrifugal filtration units with a 3.5 kDa cut-off (Millipore). The GST tag was cleaved by incubating GST-LtrK with Tev protease at a weight ratio of 100 to 1 in sonication buffer containing 1 mM TCEP and 0.5 mM EDTA, followed by elution of the GST-free LtrK. Effective removal of the GST tag was determined by visualizing bands and determining molecular weight by SDS-PAGE using a 12% (w/v) acrylamide gel followed by staining with coomassie blue solution (10% v/v acetic acid, 0.006% w/v Coomassie brilliant blue G-250 (CalbioChem)) and destaining with a solution containing 20% (v/v) methanol and 10% (v/v) acetic acid. LrtK protein sequence identity was confirmed by performing liquid chromatography mass spectrometry (LC-MS/MS) at the Bioanalytical Mass Spectrometry Facility at UNSW.

A gene encoding LtrR was commercially synthesized in a plasmid vector pJexpress404 (DNA 2.0) to produce plasmid pJexpress404-LtrR that incorporated an N- terminal 6× His tag. Overexpression was performed as for LtrK except that 1 mM IPTG was added at OD_600_ 1.0 and the cell pellet was resuspended in 40 mL of lysis buffer (20 mM HEPES pH 7.4, 250 mM NaCl and 2 tablets of complete EDTA free protease inhibitor (Roche)). The cell suspension was lysed using a French pressure cell using 1240 psi, the crude cell lysate was centrifuged at 23,000 × *g* for 30 min at 4 °C, and the supernatant filtered through an 0.45 μm syringe filter (Millipore) and loaded onto a nickel charged 5 ml HiTrap™ affinity column (GE Healthcare) that was pre-equilibrated with buffer A (20 mM HEPES pH 7.4, 500 mM NaCl). LtrR was eluted from the column using a step gradient of buffer B (20 mM HEPES pH 7.4, 500 mM NaCl, 1 M imidazole), fractions collected and LtrR identified by SDS-PAGE and concentrated as for LtrK. LtrR was further purified by size exclusion chromatography using a HiLoad 26/600 Superdex 75 prep grade gel filtration column (GE Healthcare) pre-equilibrated with gel filtration buffer (20 mM HEPES pH 7.4, 250 mM NaCl, 1 mM TCEP). The purity and confirmation of sequence identity was performed as for LtrK. All protein concentrations were determined by the Bradford method, using a Bio-Rad protein assay kit, following the manufacturer’s protocol.

### Site-directed mutagenesis and purification of mutant proteins

Amino acid replacements for histidine residues (H367, H443, H448 and H502) in LtrK (plasmid pReceiver-B03-LtrK) and aspartate residues (D54, D55 and D98) in LtrR (plasmid pJexpress404-LtrR) were generated by site-directed mutagenesis using a Phusion Site-Directed Mutagenesis kit (Thermo Scientific) according to the manufacturer’s protocol. PCR amplified DNA was circularized by incubating 20 ng of PCR product with 0.5 μl of T4 DNA ligase for 5 min, and the circularized plasmid was cloned in *E. coli* DH5α. All mutations were confirmed by DNA sequence analysis, and overexpression and purification of the mutant proteins was performed as for the wild-type LtrK and LtrR proteins, as described above.

### Differential Scanning Calorimetry

Proteins were concentrated to 3 mg ml^−1^ using 50 mM HEPES pH 8.0, 50 mM KCl, 20% glycerol, 5 mM MgCl_2_, 5 mM TCEP and 0.5 mM ATP for LtrK, and 20 mM HEPES pH 7.5 and 250 mM NaCl for LtrR. DSC melts were performed using a TA Instruments NanoDSC with a cell volume of 0.3 ml and a pressure of 3 atm. Prior to scanning, all samples and buffers were degassed under vacuum for 30 min at 4 °C. Scans were performed by increasing the temperature from 4 to 94 °C at a scan rate of either 1 °C min^−1^, 0.2 °C min^−1^ or 0.1 °C min^−1^. At least four scans were performed for each protein, including two buffer-buffer scans and two successive protein scans. Multiple rounds of melting were performed on each sample to determine whether proteins unfolded irreversibly (LtrK) or reversibly (LtrR). The data were analysed using NanoAnalyze software, and the apparent T_m_ was determined as the temperature corresponding to maximum Cp (heat capacity).

### Phosphorylation assays

Autophosphorylation assays were performed at room temperature in phosphorylation (P) buffer (50 mM HEPES pH 8.0, 50 mM KCl, 20% glycerol, 5 mM MgCl_2_, 2 mM TCEP) containing 0.2 μCi μl^−1^ [γ-^32^P]-ATP (specific activity 3000 Ci mmol^−1^; Perkin Elmer). LtrK (wild-type or mutant) and GST-LtrK proteins (1 μg each) were incubated with [γ-^32^P]-ATP in 10 μl P buffer and the reaction was terminated by adding sample buffer (106 mM Tris HCl, 141 mM Tris Base, 2% lithium dodecyl sulfate, 10% glycerol, 0.51 mM EDTA, 0.22 mM SERVA Blue G250, 0.175 mM Phenol Red pH 8.5; ThermoFisher Scientific) in a 2 to 1 volume ratio followed by heating at 95 °C for 3 min. Radioactive incorporation (autophoshorylation, kinase, autophosphatase and phosphatase) was assessed by electrophoresing samples using any kD mini-PROTEAN TGX precast gels (Bio-Rad) with visualization and quantification performed using a Fujifilm FLA5100 phosphorimager.

To assess kinase activity, phosphotransfer from LtrK-P to LtrR was performed by washing 20 μl of glutathione-agarose beads with P buffer and incubating it with 15 μg of GST-LtrK protein at room temperature for 30 min in a gravity flow column. The unbound proteins were washed off the column, and 10 μCi of [γ-^32^P]-ATP was added to the beads and autophosphorylation performed at room temperature for 30 min. The beads were thoroughly washed with P buffer to elute free ATP, and LtrR (60 μg) in 50 μl of P buffer was added to the column and the flowthrough immediately collected. Another 50 μl of buffer was passed through the column and collected with the previous flowthrough to recover all LtrR-P, and 10 μl aliquots were mixed with 3 μl of sample buffer containing 100 mM EDTA, and subjected to SDS-PAGE and autoradiography.

To determine autophosphatase activity of LtrR, the stability of LtrR-P was assessed by incubating protein in P buffer at room temperature. To determine phosphatase activity of LtrK, the dephosphorylation of LtrR-P was assessed by incubating LtrR-P with LtrK in P buffer at a molar ratio of 2 to 1 at room temperature. For both autophosphatase and phosphatase assays, a time course assessment was performed with 10 μl (~10 μg) aliquots added to 3 μl of sample buffer containing 100 mM EDTA, and the samples subjected to SDS-PAGE and autoradiography. ^32^P incorporation (activity) was quantitated as band intensity and calculated as a percentage of the total radioactivity of all samples of the protein from the time course, and plotted against time. The best exponential (for LtrR-P without LtrK) or double exponential (for LtrR-P with LtrK) fit was used to estimate model parameters including the rate constant (k), and the half-life was calculated as ln2/k.

### Temperature dependent kinase and phosphatase activities

Temperature dependent assessment of kinase and phosphatase activities were performed as described above, with temperature control achieved using a refrigerated water bath equipped with a circulator (Julabo). For autophosphorylation, 4 μg of LtrK was incubated with 0.2 μCi μl^−1^ [γ-^32^P]-ATP in 25 μl P buffer at 0, 5, 10, 15, 20, 25 or 30 °C, 6 μl aliquots withdrawn at 10 min, 0.5 h, 1 h or 2 h, and aliquots mixed with 3 μl of sample buffer, samples heated immediately at 95 °C for 3 min and subjected to SDS-PAGE and autoradiography. To assess the effect of temperature on phosphoryl group transfer from LtrK-P to LtrR, GST-LtrK bound to glutathione beads (25 μg per column) was phosphorylated in presence of [γ-^32^P]-ATP at room temperature in two gravity flow columns, as described above. After washing out the free [γ-^32^P]-ATP, one column was placed on ice at 0 °C and the other was placed at room temperature (25 °C) for 10 min. Ice cold LtrR (100 μg) in 50 μl P buffer was added to the column on ice, and an equivalent amount was warmed to room temperature for 5 min and then added to the column kept at room temperature. The flowthrough from each column was immediately collected and 10 μl samples were mixed with 3 μl of sample buffer containing 100 mM EDTA, and subjected to SDS-PAGE and autoradiography. For phosphatase activity, LtrR was phosphorylated on the column at 10 °C and 10 μl (~10 μg) of LtrR-P was incubated with LtrK in a molar ratio of 2 to 1 at 0, 5, 10, 15, 20, 25 and 30 °C for 10 min. The reaction was stopped by the addition of 5 μl of sample buffer containing 100 mM EDTA, and samples subjected to SDS-PAGE and autoradiography.

The half-life of thermal inactivation of LtrK was determined by incubating the enzyme (0.5 mg ml^−1^ final concentration in a total of 50 μl P buffer) at 10 °C or 30 °C. Aliquots (4 μl) were withdrawn at different times from 10 °C (0, 1, 5, 10, 24, 36, 48, 72, 96 h) and 30 °C (0, 5, 15, 30, 60 min), mixed with 3 μl of 0.5 μCi μl^−1^ [γ-^32^P]-ATP containing P buffer, and autophosphorylation performed at 10 °C (T_opt_) for 10 min. Note that for samples from 30 °C, aliquots were first incubated at 10 °C for 3 min to cool samples prior to the addition of [γ-^32^P]-ATP. Samples were processed for autoradiography as described above for autophosphorylation. Band intensity was used as the measure for activity, and the natural log (ln) of activity was plotted against incubation time. The inactivation constant (k_in_) was determined from the slope of the first order plot of ln activity versus time, and t_1/2_ calculated as ln2/k_in_.

### Computational analyses

Domain structure of LtrK and LtrR was analysed using the Pfam database^79^. Multiple sequence alignments were performed using ClustalW with default settings^80^. Protein homology models for LtrK were constructed using I-TASSER protein structure and function prediction server^81^ and the model was analysed using the PyMOL Molecular Graphics System, Version 1.5 Schrödinger, LLC. Domain structure organization was determined from the IMG^82^ portal (http://img.jgi.doe.gov/).

## Additional Information

**How to cite this article**: Najnin, T. *et al*. Characterization of a temperature-responsive two component regulatory system from the Antarctic archaeon, *Methanococcoides burtonii*. *Sci. Rep*. **6**, 24278; doi: 10.1038/srep24278 (2016).

## Supplementary Material

Supplementary Information
