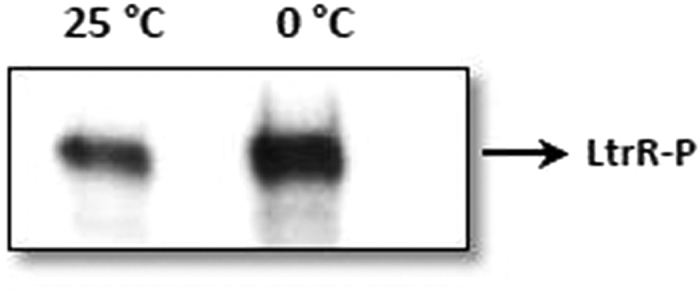# Corrigendum: Characterization of a temperature-responsive two component regulatory system from the Antarctic archaeon, *Methanococcoides burtonii*

**DOI:** 10.1038/srep27162

**Published:** 2016-06-10

**Authors:** T. Najnin, K. S. Siddiqui, T Taha, N. Elkaid, G. Kornfeld, P. M. G. Curmi, R. Cavicchioli

Scientific Reports 6: Article number: 2427810.1038/srep24278; published online: 04072016; updated: 06102016.

This Article contains an error in Figure S3; where the labels ‘0 °C’ and ‘25 °C’ are inverted. The correct Figure S3 appears below as Figure 1.

## Figures and Tables

**Figure 1:**