# Supplementary material for: Characterization of a temperature-responsive two component regulatory system from the Antarctic archaeon, Methanococcoides burtonii
Source: Sci Rep. 2016 Apr 7;6:24278. doi: 10.1038/srep24278 (PMC4823666; doi:10.1038/srep24278)
Supplement: Supplementary Information [file srep24278-s1.pdf]

Characterization of a temperature-responsive two component regulatory system from the Antarctic archaeon, *Methanococcoides burtonii*

Najnin T, Siddiqui KS, Taha, Elkaid N, Kornfeld G, Curmi PMG and Cavicchioli R

Supporting Information

Figure S1. Multiple sequence alignment of LtrK with SKs from *Bacteria* and closely related methanogens.
Figures S2. Purity of LtrK and LtrR protein preparations.
Figure S3. Effect of temperature on LtrK phosphotransfer activity.
Table S1. Organization of HTH domain in RRs from *Archaea*.
Table S2. Temperature optimum values for proteins from psychrophiles.
References

Figure S1. Multiple sequence alignment of LtrK with SKs from *Bacteria* and closely related methanogens. Multiple sequence alignments were performed using ClustalW. The conserved histidine residue H367 (labelled arrow) and the three non-conserved histidine residues, H367, H443, H448 and H502 are highlighted in the LtrK sequence in bold and yellow highlight. Amino acids identical in all sequences (*); amino acids with strong structural similarity (:); amino acids with weak structural similarity (.). A. Multiple sequence alignment of the cytoplasmic domain of LtrK with bacterial SKs that have crystal structures available for their cytoplasmic domain and share at least 35% sequence identity with LtrK. *Thermotoga maritima* HK853 (PDB: 3DGE[1]); *Streptococcus mutans* VicK (PDB: 4I5s[2]); *Bacillus subtilis* WalK (PDB: 3SL2[3]); N, G1, F, G2 and G3 blocks (red boxes). B. Alignment of full-length LtrK sequence with sequences from closely related methanogens: *Methanohalophilus mahii*, *Methanolobus tindarius*, *Methanolobus psychrophilus* and *Methanococcoides methylutens*. C. Alignment of full-length LtrK sequence with the most similar sequence available (*M. methylutens*; 75% identity), showing the lack of sequence conservation for H2 (H443), H3 (H448) and H4 (H502).

A)

```
                              H367
                               ▼
Hk853_thermotoga        TEFIANISHELRTPLTAIKAYAETIYNSLGELDLSTLKEFLEVIIDQSNHLENLLNELLD
VicK_sterptococcus      RLFVSNVSHELRTPLTSVKSYLEALDDGAL-TE-SVAPSFIKVSLDETNRMMRMITDLLS
WalK_bacillus           REFVANVSHELRTPLTSMRSYIEALEEGAW-RDPEVAPTFLNVTREETDRMIRLVNDLLQ
LtrK                    NDFVSMVSHELKTPLTAMKTSSEFLRESEC-SR-EIKEEMLDLIIRNIDRQARMVDDLLD
                          *:: :****:****::::  * : :.      .    ::.:   : ::  .:: :**.

Hk853_thermotoga        FSRLERKSLQINREKVDLCDLVESAVNAIKEFASSHNVNVLFESNVPCPVEA---YIDPT
VicK_streptococcus      LSRIDNQTSHLDVELTNFTAFMNYILDRFDQIQSQQSTNKVYEIIRDYPDKSVWIEIDTD
WalK_bacillus           LSKMDSSSDQMNSELINFNMFINKIINRHEMSQ-----GKNVTFIRDIPVKGLFVEIDPD
LtrK                    ISRIESGKMKFTPEDVNIKEIIEISLHNV----TKHAKDKSIKIMVNCPDDVPAISTDKD
                        :*:::  . ::   *   ::   :::   :.                    * .      *

                          N                                   G1             F
Hk853_thermotoga        RIRQVLLNLLNNGVKYSKKDAPDKYVKVILDEKDGGVLIIVEDNGIGIPDHAKDRIFEQF
VicK_streptococcus      KMTQVIDNILNNAIKYSPDGGKVT---ITMQTTDTQLILSISDQGLGIPKKDLPLIFDRF
WalK_bacillus           KMTQVFDNVITNAIKYSQESHKRVEFHVKQNTLYNRMTIQIKDNGIGIPVNKVDKIFDRF
LtrK                    KLIRIFVNLLTNAIKFTPEEGEVT---VIVEDHEDYLQTSIKDNGIGILEEKRDKIFDKF
                        :: ::: *::.*.:*:: .          :  :       :  :.*:*:**  .   **::*

                                    G2                    G3
Hk853_thermotoga        YRVDSSLTYEVPGTGLGLAITKEIVELHGGRIWVESEVGKGSRFFVWIPKDRAGEDNRQD
VicK_streptococcus      YRVDKARSRAQGGTGLGLAIAKEIVKQHKGFIWANSEEGEGSTFTIVLPYENDNDAIDEW
WalK_bacillus           YRVDKARARKMGGTGLGLAITKEIVEAHKGRIWASSKEGQGTSIYITLPCEVMEDEF---
LtrK                    YQVDSTATRKAGGTGLGLAIIKGIIDGQGGKIYLESETGKGSTFTFRLPKELKEDDFTEI
                        *:**.: :     ******** * *:. : * *: .*: *:*: : . :* :    :
```

B)

```
                                H367
                                 ▼
M.mahii              EFLSMVSHELKTPLTAIRTSSEFLRE-EDYDRETQEEMLDLIIRNIDRQSRMVDDLLDIS
M.tindarius          EFLSVVSHELKTPLTAMRTSSEFLRESDECDISIRRKMLDIIIRNIDRQSRMVDDLLDIS
M.psychrophilus      EFLSMVSHELKTPLTAMKTSSEFLLE-DRCDPAVRNQLLALIVRNVDRQARLVDDLLDIS
M.methylutens        DFVSMVSHELKTPLTAMKTSSEFMKE-TECDPEVKEEMLDLIIRNIDRQSRMVDDLLDIS
LtrK                 DFVSMVSHELKTPLTAMKTSSEFLRE-SECSREIKEEMLDLIIRNIDRQARMVDDLLDIS
                     :*:*:***********::*****: *     .   :.::* :*:**:***:*:********

M.mahii              RIESGRMVFKEEKVDLKEIIDNVIQMMEPMATKHGIAIERELDND-PRVKADKDKLLRVF
M.tindarius          RIESNRMKFHQETMDISKAIRCSLEMLSSNLKDKNMNVLVELPDDLSSVYTDKDKLVQVF
M.psychrophilus      RIESNRMKFNMEPVSLQEVLDHSIENIHRLSESKGVALGADISRSLPAVLADRDKLIQIF
M.methylutens        RIESGRMKFNPEDVNIREIIETSLQTVEKQAKDKGINLKVGYPQESLTIRTDKDKLIRVF
LtrK                 RIESGKMKFTPEDVNIKEIIEISLHNVTKHAKDKSIKIMVNCPDDVPAISTDKDKLIRIF
                     ****.:* *  * :.: : :   :. :    .:.: :       .   : :*:***::*

M.mahii              VNLLNNAIKFTADRGESIKIKTEETGDFVEISVIDKGIGIPENEQEKIFEKFYQVDSTSR
M.tindarius          VNLLNNAVKFSKNNGN-IRIYAQEDGDFVRISVSDDGIGMSSDELDKIFDKFYQIDSTST
M.psychrophilus      VNLLNNAVKFTQRGGS-VTVLAQESGDNIEVIVSDTGIGIDPSHAEHIFDKFYQIDSTST
M.methylutens        VNLLTNAIKFTPHEGD-VEISVTDTGDNIQASIKDNGIGIPPEKRDKIFDKFYQVDSTAT
LtrK                 VNLLTNAIKFTPEEGE-VTVIVEDHEDYLQTSIKDNGIGILEEKRDKIFDKFYQVDSTAT
                     ****.**:**:   *. : : . :  * :.  : * ***:  .. ::**:****:***:

M.mahii              RKVGGSGLGLAIIKGIIEGHGGSIRVKSEPGVGSTFVFTLRKWEE----------
M.tindarius          RKVGGSGLGLSIVKGIIEGQGGTISATSEPGNGSTFVFTLKKSM-----------
M.psychrophilus      RTAGGCGLGLAITKGLVEGMNGSIRVESEPGIGSRFIVTLKKAGI----------
M.methylutens        RKAGGTGLGLAIIKGIIDGQGGNIFVESDNGKGSTFTFRLPKELKEEDFTEIEQH
LtrK                 RKAGGTGLGLAIIKGIIDGQGGKIYLESETGKGSTFTFRLPKELKEDDFTEIEGT
                     *..** ****:* **:::* .*.*   *: * ** * . * *
```

C)

```
                             H367
                              ▼
M.methylutens        FVSMVSHELKTPLTAMKTSSEFMKETECDPEVKEEMLDLIIRNIDRQSRMVDDLLDISRI
LtrK                 FVSMVSHELKTPLTAMKTSSEFLRESECSREIKEEMLDLIIRNIDRQARMVDDLLDISRI
                     **********************::*:**. *:***************:************

M.methylutens        ESGRMKFNPEDVNIREIIETSLQTVEKQAKDKGINLKVGYPQESLTIRTDKDKLIRVFVN
LtrK                 ESGKMKFTPEDVNIKEIIEISLHNVTKHAKDKSIKIMVNCPDDVPAISTDKDKLIRIFVN
                     ***:***.******:**** **:.* *:****.*:: *. *::  :* ********:***

M.methylutens        LLTNAIKFTPHEGDVEISVTDTGDNIQASIKDNGIGIPPEKRDKIFDKFYQVDSTATRKA
LtrK                 LLTNAIKFTPEEGEVTVIVEDHEDYLQTSIKDNGIGILEEKRDKIFDKFYQVDSTATRKA
                     **********.**:* : * *  * :*:*********  *********************

M.methylutens        GGTGLGLAIIKGIIDGQGGNIFVESDNGKGSTFTFRLPKELKEEDFTEIEQH
LtrK                 GGTGLGLAIIKGIIDGQGGKIYLESETGKGSTFTFRLPKELKEDDFTEIEGT
                     *******************:*::**:.****************:******
```

Figure S2. Purity of LtrK and LtrR protein preparations. The fractions from each purification step were analysed on a SDS-polyacrylamide gel (12%) with Coomassie brilliant blue staining. A. SDS-PAGE of different fractions during LtrK purification. Lanes: 1, soluble fraction of sonicated lysate; 2 & 3, flowthrough fractions; 4, eluted GST-LtrK by 20 mM glutathione; 5, GST tag cleaved LtrK; 6, Purified LtrK after cleavage reaction; 7, Eluted GST tag obtained after cleavage; 8, protein molecular weight standard. B. SDS-PAGE of different fractions during LtrR purification. Lanes: 1, eluted 6×His LtrR with 100 mM imidazole; 2, flowthrough fraction of 20 mM imidazole wash; 3, soluble fraction of sonicated lysate; 4, protein molecular weight standard.

A)

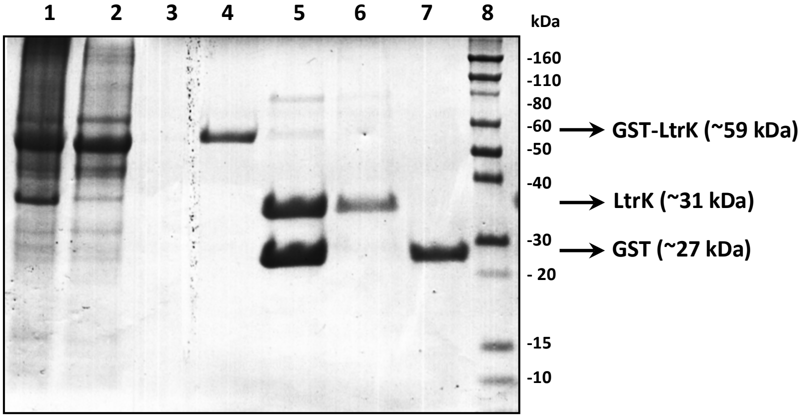
1
2
3
4
5
6
7
8
kDa
-160
-110
-80
-60
-50
-40
-30
- 20
-15
-10
GST-LtrK (~59 kDa)
LtrK (~31 kDa)
GST (~27 kDa)

B)

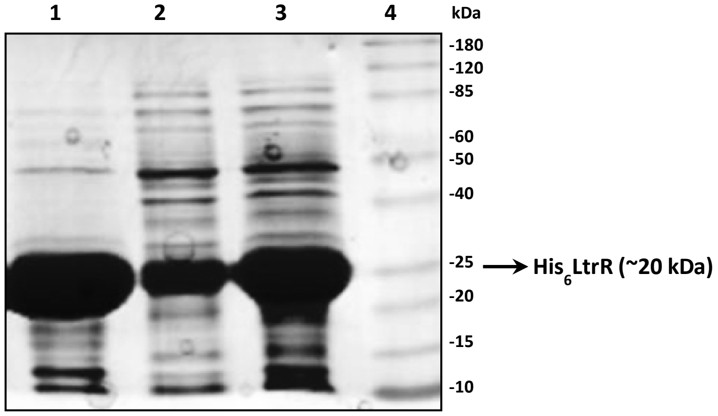
1
2
3
4
kDa
-180
-120
-85
-60
-50
-40
-25
-20
-15
-10
His6LtrR (~20 kDa)

Figure S3. Effect of temperature on LtrK phosphotransfer activity. GST-LtrK (25 μg) bound to glutathione beads was phosphorylated in presence of [γ-32P]-ATP at room temperature for 30 min, free [γ-^{32}P]-ATP washed off and one column was placed on ice (0 °C) and the other was placed at room temperature (25 °C) for 10 min. Ice cold LtrR (100 μg) was passed through the cold column and an equivalent amount of LtrR was warmed to room temperature for 5 min and then added to the column kept at room temperature. LtrR-P was collected in the flowthrough and 10 μl samples were added to 3 μl of 100 mM EDTA containing sample buffer and analysed by gel-phosphorimaging.

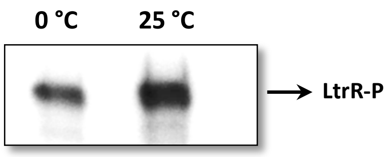
0 °C
25 °C
LtrR-P

Table S1. Organization of HTH domain in RRs from *Archaea*.

Archaea	N-terminal	C-terminal
**Halophilic**		
Halonotius sp. J07HN		J07HN4v2_01675, J07HN4v2_00780, J07HN4v2_02997, J07HN6v2_01307
Halorubrum aidingense		C461_00607
Halorubrum lacusprofundi		Hlac_3274
Halobiforma lacisalsi		C445_12891
Halopiger djelfamassiliensis		Ga0036365_12412, Ga0036365_12651, Ga0036367_103616
Haloterrigena turkmenica		Htur_0128, Htur_0348, Htur_0709
Haloquadratum walsbyi		Hqrw_3103
Haloferax volcanii		HVO_1357, HVO_B0272
Haloarcula marismortui	rrnB0301	pNG7159, pNG7223
Halopiger xanaduensis		Halxa_3599
Halovivax ruber		Halru_0454
Halobacterium sp. DL1	HalDL1_2148, HalDL1_1858	HalDL1_2066
Halostagnicola larsenii		Halla_0473
Halalkalicoccus jeotgali	HacjB3_0162	HacjB3_04505, HacjB3_00310, HacjB3_18828
Halorhabdus utahensis		Huta_1740, Huta_0317
Halohasta litchfieldiae tADL		halTADL_2543
Natrinema pallidum		C487_17780
Natrinema pellirubrum		C488_09871
Natrinema versiforme		C489_01256
Natronolimnobius innermongolicus		C493_05780
Natronorubrum tibetense		C496_05962
Natrialba hulunbeirensis		C483_14902
Natrialba magadii	Nmag_2044	Nmag_1012, Nmag_2147, Nmag_2692, C500_06936
Natrialba taiwanensis		C484_14808
Natrialba aegyptia		C480_05466
Natrialba chahannaoensis		C482_19154
Natronobacterium gregoryi		Natgr_1657
Natronococcus amylolyticus		C491_04490
Natronomonas pharaonis Gabara		NP0654A
**Psychrophilic & mesophilic methanogens**		
Methanococcoides methylutens	Ga0070849_114114	
Methanohalophilus mahii	Mmah_0822	
Methanolobus psychrophilus	Mpsy_1252	
Methanosarcina barkeri	Mbar_A2896	
Methanosarcina mazei	MM2351	
Methanococcoides burtonii	Mbur_0695 (LtrK)	

Table S2. Temperature optimum values for proteins from psychrophiles.

Organism	Protein	T_{opt}	Reference No.
Bacteria			
Antarctic bacterium	Citrate synthase	31 °C	4
Antarctic *Pseudomonas* sp.	Phosphoglycerate Kinase	35 °C	5
Antarctic *Pseudomonas syringae*	RNA polymerase	37 °C	6
Antarctic seawater bacterium	Alkaline phosphatase	25 °C	7
Antarctic seawater bacterium	DNA ligase	16 °C	8
Arctic sea shore sediment bacterium	Esterase	30 °C	9
Bacillus globisporus	Adenylate kinase	35 °C	10
Bacillus TA41	Subtilisin	40 °C	11
Carnobacterium piscicola BA	β-galactosidase	30 °C	12
Chlamys islandica	Chlamysin	22 °C	13
Colwellia psychrerythraea	Aminopeptidase	39 °C	14
Colwellia psychrerythraea	Phenylalanine hydroxylase	25 °C	15
Colwellia maris	Malate synthase	45 °C	16
Colwellia maris	Isocitrate lyase	20 °C	16
Cold sea sediment sample bacterium	Lipase	25 °C	17
Cytophaga sp.	Valine dehydrogenase	20 °C	18
Desulfotalea psychrophila	Isocitrate dehydrogenase	45 °C	19
Marine psychrophile strain PA-43	Serine protease	58 °C	20
Micrococcus sp.	Pollulanase (extracellular)	50 °C	21
Moraxella sp.	Alcohol dehydrogenase	25 °C	22
Pseudoalteromonas haloplanktis	α-amylase	28 °C	23
P. haloplanktis	Cellulase	40 °C	24
P. haloplanktis	Xylanase	35 °C	25
P. haloplanktis	Pectate lyase (extracellular)	30 °C	26
P. haloplanktis	Aspartate amino transferase	64 °C	27
Pseudomonas strain DY-A	Serine Alkaline protease	40 °C	28
Sphingomonas paucimobilis (Antarctic marine bacterium)	Extracellular metalloprotease	25 °C	29
Archaea			
Cenarchaeum symbiosum	DNA polymerase	40 °C	30
Methanococcoides burtonii	EF2	34 °C	31
Eucarya			
Antarctic fish	Lactate dehydrogenase	50 °C	32
Fish liver	Imidase	55 °C	33
Psychrophilic green alga	Argininosuccinate lyase	37 °C	34
Sclerotinia borealis	Polygalacturonase (extracellular)	45 °C	35

*When data were available for multiple enzymes of the same type (e.g. α-amylases from different psychrophiles), data were included for the enzyme with the lowest T_{opt}.

References

1. Marina, A., Waldburger, C. D. & Hendrickson, W. A. Structure of the entire cytoplasmic portion of a sensor histidine-kinase protein. *EMBO J.* **24,** 4247-4259 (2005).
2. Wang, C. *et al.* Mechanistic insights revealed by the crystal structure of a histidine kinase with signal transducer and sensor domains. *PLoS Biol.* **11,** e1001493 (2013).
3. Celikel, R., Veldore, V. H., Mathews, I., Devine, K. M. & Varughese, K. I. ATP forms a stable complex with the essential histidine kinase WalK (YycG) domain. *Acta Crystallogr. D Biol. Crystallogr.* **68,** 839-845 (2012).
4. Russell, R. J., Gerike, U., Danson, M. J., Hough, D. W. & Taylor, G. L. Structural adaptations of the cold-active citrate synthase from an Antarctic bacterium. *Structure* **6,** 351-361 (1998).
5. Bentahir, M. *et al.* Structural, kinetic, and calorimetric characterization of the cold-active phosphoglycerate kinase from the Antarctic *Pseudomonas* sp. TACII18. *J. Biol. Chem.* **275,** 11147-11153 (2000).
6. Uma, S., Jadhav, R., Kumar, G. S., Shivaji, S. & Ray, M. A RNA polymerase with transcriptional activity at 0 C from the Antarctic bacterium *Pseudomonas syringae. FEBS Lett.* **453,** 313-317 (1999).
7. Kobori, H., Sullivan, C. W. & Shizuya, H. Heat-labile alkaline phosphatase from Antarctic bacteria: rapid 5′ end-labeling of nucleic acids. *Proc. Natl. Acad. Sci. USA* **81,** 6691-6695 (1984).
8. Georlette, D. *et al.* Structural and functional adaptations to extreme temperatures in psychrophilic, mesophilic, and thermophilic DNA ligases. *J. Biol. Chem.* **278,** 37015-37023 (2003).
9. Jeon, J. H. *et al.* Cloning and characterization of a new cold-active lipase from a deep-sea sediment metagenome. *Appl. Microbiol. Biotechnol.* **81,** 865-874 (2009).
10. Bae, E. & Phillips, G. N. Structures and analysis of highly homologous psychrophilic, mesophilic, and thermophilic adenylate kinases. *J. Biol. Chem.* **279,** 28202-28208 (2004).
11. Davail, S., Feller, G., Narinx, E. & Gerday, C. Cold adaptation of proteins. Purification, characterization, and sequence of the heat-labile subtilisin from the antarctic psychrophile *Bacillus* TA41. *J. Biol. Chem.* **269,** 17448-17453 (1994).

12. Coombs, J. M. & Brenchley, J. E. Biochemical and phylogenetic analyses of a cold-active β-galactosidase from the lactic acid bacterium *Carnobacterium piscicola* BA. *Appl. Environ. Microbiol.* **65,** 5443-5450 (1999).
13. Nilsen, I. W. *et al.* Protein purification and gene isolation of chlamysin, a cold-active lysozyme-like enzyme with antibacterial activity. *FEBS Lett.* **464,** 153-158 (1999).
14. Huston, A. L., Haeggström, J. Z. & Feller, G. Cold adaptation of enzymes: Structural, kinetic and microcalorimetric characterizations of an aminopeptidase from the Arctic psychrophile *Colwellia psychrerythraea* and of human leukotriene A 4 hydrolase. *Biochim. Biophys. Acta*. **1784,** 1865-1872 (2008).
15. Leiros, H.-K. S. *et al.* Structure of phenylalanine hydroxylase from Colwellia psychrerythraea 34H, a monomeric cold active enzyme with local flexibility around the active site and high overall stability. *J. Biol. Chem.* **282,** 21973-21986 (2007).
16. Watanabe, S., Takada, Y. & Fukunaga, N. Purification and characterization of a cold-adapted isocitrate lyase and a malate synthase from *Colwellia maris*, a psychrophilic bacterium. *Biosci. Biotechnol. Biochem*. **65,** 1095-1103 (2001).
17. Jeon, J. H., Kim, J.-T., Kang, S. G., Lee, J.-H. & Kim, S.-J. Characterization and its potential application of two esterases derived from the arctic sediment metagenome. *Mar. Biotechnol.* **11,** 307-316 (2009).
18. Oikawa, T., Yamanaka, K., Kazuoka, T., Kanzawa, N. & Soda, K. Psychrophilic valine dehydrogenase of the antarctic psychrophile, *Cytophaga* sp. KUC-1. *Eur. J. Biochem.* **268,** 4375-4383 (2001).
19. Fedøy, A.-E., Yang, N., Martinez, A., Leiros, H.-K. S. & Steen, I. H. Structural and functional properties of isocitrate dehydrogenase from the psychrophilic bacterium *Desulfotalea psychrophila* reveal a cold-active enzyme with an unusual high thermal stability. *J. Mol. Biol.* **372,** 130-149 (2007).
20. Irwin, J. A., Alfredsson, G. A., Lanzetti, A. J., Gudmundsson, H. M. & Engel, P. C. Purification and characterisation of a serine peptidase from the marine psychrophile strain PA-43. *FEMS Microbiol. Lett.* **201,** 285-290 (2001).
21. Kimura, T. & Horikoshi, K. Characterization of pullulan-hydrolysing enzyme from an alkalopsychrotrophic *Micrococcus* sp. *Appl. Microbiol. Biotechnol.* **34,** 52-56 (1990).
22. Tsigos, I., Velonia, K., Smonou, I. & Bouriotis, V. Purification and characterization of an alcohol dehydrogenase from the Antarctic psychrophile *Moraxella* sp. TAE123. *Eur. J. Biochem.* **254,** 356-362 (1998).

23. D'Amico, S., Gerday, C. & Feller, G. Temperature adaptation of proteins: engineering mesophilic-like activity and stability in a cold-adapted α-amylase. *J. Mol. Biol.* **332,** 981-988 (2003).
24. Garsoux, G., Lamotte, J., Gerday, C. & Feller, G. Kinetic and structural optimization to catalysis at low temperatures in a psychrophilic cellulase from the Antarctic bacterium *Pseudoalteromonas haloplanktis*. *Biochem. J.* **384,** 247-253 (2004).
25. Collins, T., Meuwis, M.-A., Gerday, C. & Feller, G. Activity, stability and flexibility in glycosidases adapted to extreme thermal environments. *J. Mol. Biol.* **328,** 419-428 (2003).
26. Tuyen, H., Helmke, E. & Schweder, T. Cloning of two pectate lyase genes from the marine Antarctic bacterium *Pseudoalteromonas haloplanktis* strain ANT/505 and characterization of the enzymes. *Extremophiles* **5,** 35-44 (2001).
27. Birolo, L. *et al.* Aspartate aminotransferase from the Antarctic bacterium *Pseudoalteromonas haloplanktis* TAC 125. *J Biochem*. **267,** 2790-2802 (2000).
28. Zeng, R., Zhang, R., Zhao, J. & Lin, N. Cold-active serine alkaline protease from the psychrophilic bacterium *Pseudomonas* strain DY-A: enzyme purification and characterization. *Extremophiles* **7,** 335-337 (2003).
29. Turkiewicz, M., Gromek, E., Kalinowska, H. & Zielińska, M. Biosynthesis and properties of an extracellular metalloprotease from the Antarctic marine bacterium *Sphingomonas paucimobilis*. *J. biotechnol.* **70,** 53-60 (1999).
30. Schleper, C., Swanson, R. V., Mathur, E. J. & DeLong, E. F. Characterization of a DNA polymerase from the uncultivated psychrophilic archaeon *Cenarchaeum symbiosum*. *J. Bacteriol.* **179,** 7803-7811 (1997).
31. Thomas, T. & Cavicchioli, R. Effect of temperature on stability and activity of elongation factor 2 proteins from Antarctic and thermophilic methanogens. *J. Bacteriol.* **182,** 1328-1332 (2000).
32. Coquelle, N., Fioravanti, E., Weik, M., Vellieux, F. & Madern, D. Activity, stability and structural studies of lactate dehydrogenases adapted to extreme thermal environments. *J. Mol. Biol.* **374,** 547-562 (2007).
33. Huang, C.-Y. & Yang, Y.-S. A novel cold-adapted imidase from fish *Oreochromis niloticus* that catalyzes hydrolysis of maleimide. *Biochem. Biophys. Res. Commun.* **312,** 467-472 (2003).

34. Loppes, R., Devos, N., Willem, S., Barthélemy, P. & Matagne, R. F. Effect of temperature on two enzymes from a psychrophilic chloromonas (*Chlorophyta*). *J. Phycol.* **32,** 276-278 (1996).

35. Takasawa, T. *et al.* Polygalacturonase isolated from the culture of the psychrophilic fungus *Sclerotinia borealis*. *Can. J. Microbiol.* **43,** 417-424 (1997).
